# Synthetic and minimalist vectors for *Agrobacterium tumefaciens*-mediated transformation of fungi

**DOI:** 10.1590/1678-4685-GMB-2018-0221

**Published:** 2019-06-13

**Authors:** Luísa Czamanski Nora, Relber Aguiar Gonçales, Leonardo Martins-Santana, Beatriz Henriques Ferreira, Fernando Rodrigues, Rafael Silva-Rocha

**Affiliations:** 1 Systems and Synthetic Biology Laboratory, Departmento de Biologia Celular e Molecular Biology e Bioagentes Patogênicos, Faculdade de Medicina de Ribeião Preto, Universidade de São Paulo, Ribeirão Preto, SP, Brazil; 2 Immunochemistry and Glycobiology Laboratory, Departmento de Biologia Celular e Molecular Biology e Bioagentes Patogênicos, Faculdade de Medicina de Ribeião Preto, Universidade de São Paulo, Ribeirão Preto, SP, Brazil; 3 Life and Health Sciences Research Institute (ICVS), School of Health Sciences, University of Minho, Braga, Portugal

**Keywords:** synthetic biology, fungi, Agrobacterium tumefaciens, transformation, vectors.

## Abstract

We present a collection of minimalist binary vectors for transformation through ATMT applicable to several fungi species. pLUO plasmid binary vectors consist of a reporter module containing fluorescent proteins, mCherry or eGFP, flanked by a multiple cloning site and a transcription terminator site. They also present a synthetic gene allowing resistance to Hygromicin B flanked by alternate promoters, one for yeast and another for filamentous fungi. Left and right borders were added for *Agrobacterium tumefaciens* recognition, and a minimal broad-host range RK2 replication origin. Transformation was validated in the pathogenic fungus *Paracoccidioides lutzii*. Hence, we developed an efficient and reliable molecular tool for fungal transformation: minimalist, synthetic, modular, and available in four different versions, and these can still be readily modified using a few primers and few cloning steps.

## Introduction

Fungi are organisms comprising a universe that has not been fully explored by mankind(Leigh *et al.*, 2003), but have been extensively studied because of their huge impact in everyday life and their endless applications in industry, such as production of biofuels (Glass *et al.*, 2013), foods and feedstock (Bhat, 2000), human therapeutics (Ward, 2012), among many others. Likewise, even greater efforts are being engaged in studying their pathogenicity (Almeida *et al.*, 2007; Teixeira *et al.*, 2013). Tools that can provide a better understanding of the molecular mechanisms that control gene expression in those organisms are useful, not only for shedding light on their functioning, but also because it can be used for genetic engineering and delivery of products. Synthetic Biology is an ever-growing field responsible for building new genetic circuits with known biological parts, and a great amount of the challenge in this area is in finding minimal synthetic vectors that provide a desirable setting for this cycle of re-designing parts (Silva-Rocha *et al.*, 2013; Pasin *et al.*, 2017). Fungi and synthetic biology are a promising combination that is opening brand-new doors for science, however, there is still plentiful of work to be done (Amores *et al.*, 2016). In the pursuit of overcoming the lack of tools for fungal studies, we developed the pLUO vectors, a collection of minimal and versatile binary plasmid vectors for *A. tumefaciens*-mediated transformation (ATMT).

**Figure 1:**
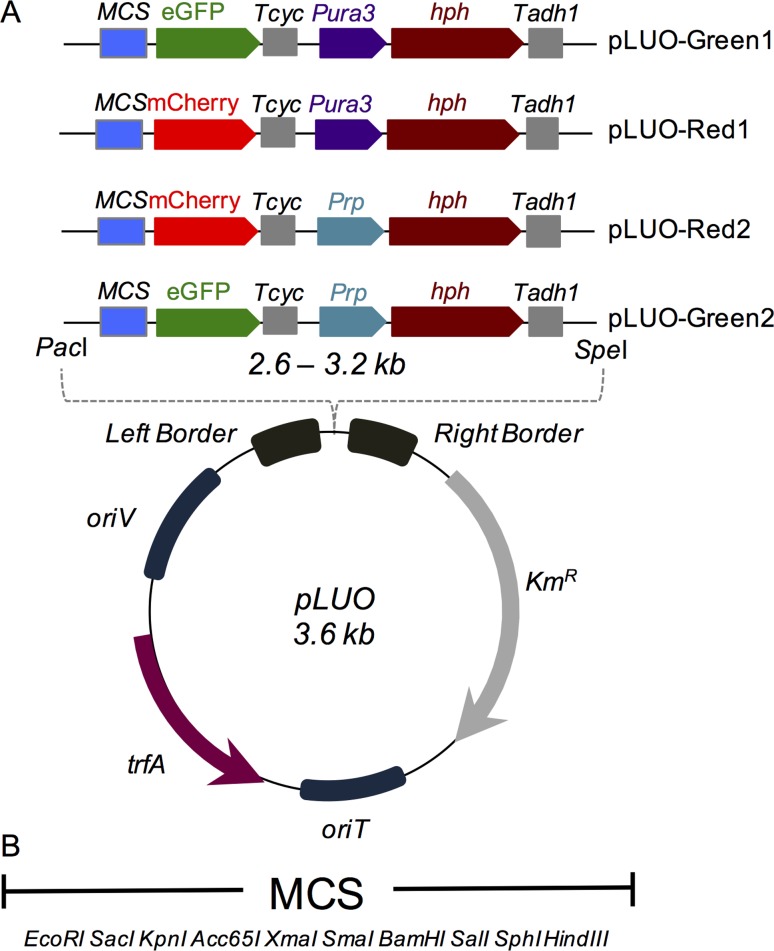
Representative scheme of the minimal binary vector for fungi transformation through ATMT. (A) Design of the plasmid showing all the minimal modules that compose it, including the four versions of the expression *cassette* that were constructed. pLUO-Green1 (with eGFP) and pLUO-Red1 (with mCherry) have a *Pura3* promoter for *hph* expression, while pLUO-Green2 and pLUO-Red2 uses *Prp2* from *T. reesei*. (B) Representation of the restriction enzymes available in the MCS for several cloning options.

The pLUO vectors were constructed using minimal essential parts so that they could be reduced in size while still keeping their functionality. This was achieved by employing the pGLR2 plasmid as vector backbone (Benedetti *et al.*, 2012) that is also minimum and presents a broad host range RK2 origin of replication, so it replicates in *E. coli* and in *A. tumefaciens*. pLUO vectors present a multiple cloning site (MCS) with 11 different restriction sites for several cloning options, so any given promoter can be placed to modulate a red (mCherry) or a green (eGFP) reporter protein. The selection marker is a synthetic, codon-optimized, and free from restriction sites gene, allowing resistance to Hygromicin B (*hph*) flanked by two different optimized promoters – so one can choose to transform it into yeast using *Pura3* or into filamentous fungi using *Prp2* – and a terminator (*Tadh1*). Two regions of 25 direct imperfect repeats were added at both ends of this *cassette*, the left and right borders, so that *A. tumefaciens* can recognize, nick and transfer the DNA from the binary vector to the host (Figure 1). The method of ATMT for fungi has been widely used for a long time due to its high yield of positive transformants (Michielse *et al.*, 2008).

**Figure 2:**
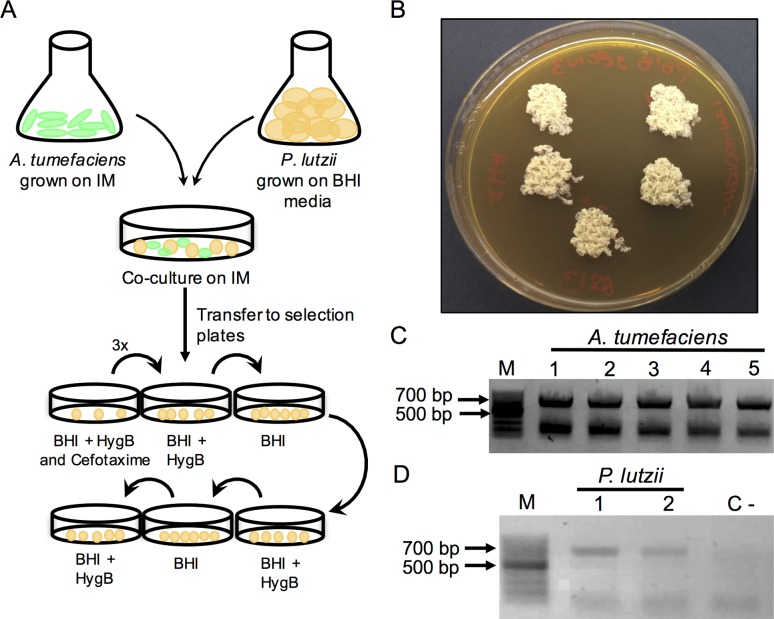
Validation of pLUO vector in *P. lutzii*. (A) Representative image of experiment workflow. The first selection plate contains Cefotaxime 100 μg/mL to kill the remaining *A. tumefaciens* colonies. Afterwards, fungal colonies are selected on BHI containing only Hygromicin B for three rounds, and then alternating media with or without Hygromicyn until reaching nine rounds of mitotic stability. (B) *P. lutzii* colonies transformed with pLUO vector after reaching mitotic stability. (C) Eletrophoresis gel showing the colony PCR reactions from *A. tumefaciens* as a positive control with the *hph* gene amplified by its primers (resulting in a band of 705 base pairs; the lower band is an unespecific one that always appears for *A. tumefaciens*). (D) Eletrophoresis gel showing the PCR reactions of *P. lutzii* transformants. M, molecular ladder; and C, negative control of the reaction.

The validation of pLUO vectors was performed in the fungus *P. lutzii*, a dimorphic human opportunistic pathogen. Most of the vectors used for its molecular studies contained more than 15,000 base pairs (Almeida *et al.*, 2007), making pLUO a desirable substitute, since it comprises only 6 kb. Six rounds of selection were performed as established to reach mitotic stability. Three more round were done to verify stability (Figure 2A) with the expression *cassette* (Figure 2B), for a total of nine rounds. The electrophoresis gel shows that the transformants were positive for the *hph* gene, which proves that transformation was successful (Figure 2C,D). Henceforward, this vector would be applicable as an efficient method to study gene expression in this pathogenic organism. The expression *cassette* tested contains an eGFP reporter and *Pura3* modulating *hph* – the following versions were all adapted by overlapping PCR reactions. Thus, expansion of the modules can be performed by using the primers provided (Table 1), or variations, when the aim is to build additional versions that suit other target organisms. *P. lutzii* was used as a model to prove the functionality of this vector, but studies in other strains will be further developed using this collection of vectors.

**Table 1:** Primers used in this study.

Name	Sequence (5’- 3’)	Target DNA
5’_GFP_*HindIII*	GCGCAAGCTTCGGTATCGATCATGAGTAAAG	pMCB17-apx
3’_GFP	TTAAGCCGGCGCGCC	pMCB17-apx
5’_*Tcyc*_GFP	GGCGCGCCGGCTTAACTCCTCCCACATCCGC	*S. cerevisae*
3’_*Tcyc*_*SpeI*_*BamHI*	GCGCGGATCCACTAGTAAGCCTTCGAGCGTCCC	*S. cerevisae*
5’_*hph*	CTGACACTAGCGCCACC	pGL4.14
3’_*hph*	GTTTAAACTCGACCTACCTCC	pGL4.14
5’_*Pura3*_*XbaI*	GCGCTCTAGAGTGCACCATACCACAGC	pRS426^14^
3’_*Pura3*_*hph*	TGGCGCTAGTGTCAGTGAGATTTATCTTCGTTTCCTGC	pRS426^14^
5’_*Tadh1*_*hph*	AGGTCGAGTTTAAACGGTAGATACGTTGTTGACAC	*S. cerevisae*
3’_*Tadh1*_*SpeI*_*EcoRI*	GCGCGAATTCACTAGTGTGGTCAATAAGAGCGACC	*S. cerevisae*
5’_LB_GFP_*PacI*	GCGCTTAATTAATGGCAGGATATATTGTGGTGTAAACATAACAATTTCACACAGGACCTAGG	pLUO
3’_RB_*Tadh1*_*SpeI*	GCGCACTAGTGTTTACCCGCCAATATATCCTGTCAGTGGTCAATAAGAGCGACC	pLUO
5’_*Tcyc*	CTCCTCCCACATCCGC	pLUO
3’_*Tcyc*	AAGCCTTCGAGCGTCC	pLUO
5’_*HindIII*_mCherry	GCGCAAGCTTGGTATGGTGAGCAAGGGC	pMR1^17^
3’_mCherry_*Tcyc*	GGTTAGAGCGGATGTGGGAGGAGTTACTTGTACAGCTCGTCC	pMR1^17^
5’_*Tcyc*_*Prp2*	GGTTTTGGGACGCTCGAAGGCTTCGGCTGCGTGAACAGACG	*T. reesei*
3’_*Prp2*_*hph*	GGTGGCGCTAGTGTCAGGTGGTTTGAGTTGGGTTGAGATAGG	*T. reesei*

Sites for restriction enzymes are underlined.

For the construction of the plasmids, the eGFP protein was amplified from pMCB17-apx (Fernandez-Abalos *et al.*, 1998) and its terminator was from the *cyc1* gene from *S. cerevisae* genome. These fragments were fused using overlapping PCR with Phusion^®^ High Fidelity DNA Polymerase (NEB), adding restriction sites for *Hind*III, *Spe*I and *Bam*HI. The fragment was cloned into the high-copy number vector pUC19 (Yanisch-Perron *et al.*, 1985) using *Hind*III and *Bam*HI for the digestion reaction, and then transformed into chemocompetent *E. coli* DH10B. The *hph* gene is from pGL4.14 (Promega) and its terminator was amplified from the *adh1* gene of *S. cerevisae*. The variations of promoters modulating the *hph* gene were the following: the *ura3* promoter (*Pura3*) for yeast was amplified from the pRS426 shuttle vector (Christianson *et al.*, 1992), and *Prp2* for filamentous fungi was amplified from the *T. reesei* genome (He *et al.*, 2013). For construction of the yeast *cassette*, the three fragments – *Pura3*, *hph*, *Tadh1* – were fusioned by overlapping PCR, and the restriction sites for *Xba*I, *Spe*I and *Eco*RI were added by primers. Then, this fragment was digested with *Xba*I and *Eco*RI and inserted into pUC19 containing the reporter module eGFP_*Tcyc*, digested with the same enzymes. The entire expression *cassette* was amplified from pUC19 using primers to include the borders and, then, cloned into the pGLR2 vector using *Pac*I and *Spe*I*.* The variations in the *cassette* were all built by a few reactions of overlapping PCR using the first one as template. All enzymes used in this work were from New England Biolabs and all primers are shown in Table 1.

The strain of *A. tumefaciens* was LBA1100 with a disarmed octopine-type pTiB6 plasmid (Menino *et al.*, 2012) and was transformed with the vectors by electroporation. *P. lutzii* transformation through ATMT was done as described in Menino *et al.* (2012). Colonies of *P. lutzii* were randomly selected and plated into solid BHI media containing 75 μg/mL Hygromicin B three consecutive times. Subsequently, they were serially transferred to media with or without the selection marker for three times each, totalizing nine rounds of selection, growing for 15-20 days between each round. All plasmids and sequences can be made available upon request to the authors.
